# Supplementary material for: Early Changes in DCE-MRI Biomarkers May Predict Survival Outcomes in Patients with Advanced Hepatocellular Carcinoma after Sorafenib Failure: Two Prospective Phase II Trials
Source: Cancers (Basel). 2021 Oct 1;13(19):4962. doi: 10.3390/cancers13194962 (PMC8508238; doi:10.3390/cancers13194962)
Supplement: Supplementary file 1 [file cancers-13-04962-s001.zip › cancers-1313279-supplementary.pdf]

Supplementary file

Table S1. Patient clinical and imaging characteristics according to treatment response

	PR	SD	PD	P value
**Number**	8	35	31	
**Age, year (mean, SD)**	60.9 (10.8)	60.5 (12.9)	59.2 (11.1)	0.885
**Sex (women)**	1 (12.5)	5 (14.3)	4 (12.9)	0.983
**Tumor size (cm^2, mean, SD)**	85.4 (65.6)	85.9 (56)	76.1 (42)	0.729
**Etiology**				
**Hepatitis B**	4 (50.0)	24 (68.6)	23 (74.2)	0.419
**Hepatitis C**	3 (37.5)	6 (17.1)	5 (16.1)	0.362
**Alcoholic**	3 (37.5)	1 (2.9)	2 (6.5)	0.005
**ECOG**				0.078
**0**	0 (0)	13 (37.1)	7 (22.6)	
**1**	8 (100.0)	22 (62.9)	24 (77.4)	
**AFP > 400 ng/mL**	7 (87.5)	16 (45.7)	18 (58.1)	0.093
**Liver cirrhosis**	7 (87.5)	26 (74.3)	22 (71)	0.542
**Vascular invasion**	6 (75.0)	18 (51.4)	17 (54.8)	0.479
**Extrahepatic spread**	7 (87.5)	29 (82.9)	27 (87.1)	0.872
**Child-Pugh**				0.03
**5**	3 (27.5)	29 (82.9)	23 (74.2)	
**6**	5 (62.5)	6 (17.1)	8 (25.8)	
**Prior therapy**				
**Surgery**	2 (25.0)	20 (57.1)	14 (45.2)	0.228
**Ablation**	2 (25.0)	11 (31.4)	6 (19.4)	0.533
**TACE**	7 (87.5)	27 (77.1)	26 (83.9)	0.696

Note. – Unless otherwise indicated, data are numbers, and data in parenthesis are percentages. PR = partial response, SD = stable disease, PD = progressive disease, AFP = alpha-fetoprotein, TACE = transarterial chemoembolization, ECOG = Eastern Cooperative Oncology Group.

Table S2. Comparison of changes of DCE-MRI parameters and cutoff values by disease control rate (DCR)

DCE-MRI parameters	PR + SD	PD	P value	Cutoff values	AUROC	Sensitivity	Specificity
**∆Peak_D3 (%)**	-3.0 (-12.1, 3.7)	-6.4 (-13.3, 4.0)	0.722	-5.8	0.52	0.55	0.65
**∆AUC_D3 (%)**	-11.20 (-37.1, 11.9)	-12. (-31.9, 18.8)	0.969	-54.6	0.5	0.97	0.16
**∆K^{trans}_D3 (%)**	-30.20 (-60.6, 14.9)	0.00 (-33.1, 44.9)	0.075	-9.3	0.62	0.58	0.67
**∆Peak _D14 (%)**	-3.1 (-16.3, 2.6)	-7.0 (-15.1, 5.8)	0.932	4.59	0.51	0.33	0.83
**∆AUC_D14 (%)**	-25.5 (-42.3, 8.4)	-15.3 (-49.4, 26.6)	0.656	-21.1	0.53	0.67	0.52
**∆K^{trans}_D14 (%)**	-44.6 (-61.7, -14.9)	-3.4 (-40.9, 95.4)	0.01	-11.8	0.68	0.63	0.76

Note. – The data are expressed as median and interquartile range.

* *P* value indicates a significant difference. Youden index is used for seeking the best cutoff value for ROC Curve.

PR = partial response, SD = stable disease, PD = progressive disease, AUROC = area under the receiver operating characteristic.
